# Determination of the membrane topology of PORCN, an O-acyl transferase that modifies Wnt signalling proteins

**DOI:** 10.1098/rsob.200400

**Published:** 2021-06-30

**Authors:** Lisa M. Galli, Marc O. Anderson, J. Gabriel Fraley, Luis Sanchez, Raymund Bueno, David N. Hernandez, Eva U. Maddox, Vishwanath R. Lingappa, Laura W. Burrus

**Affiliations:** ^1^ Department of Biology, San Francisco State University, 1600 Holloway Avenue, San Francisco, CA 94132, USA; ^2^ Department of Chemistry and Biochemistry, San Francisco State University, 1600 Holloway Avenue, San Francisco, CA 94132, USA; ^3^ Prosetta Biosciences, 670 5th Street, San Francisco, CA 94107, USA

**Keywords:** Porcupine, Wnt, membrane topology, palmitoylation, MBOAT, O-acyl transferase

## Abstract

Wnt gradients elicit distinct cellular responses, such as proliferation, specification, differentiation and survival in a dose-dependent manner. Porcupine (PORCN), a membrane-bound O-acyl transferase (MBOAT) that resides in the endoplasmic reticulum, catalyses the addition of monounsaturated palmitate to Wnt proteins and is required for Wnt gradient formation and signalling. In humans, PORCN mutations are causal for focal dermal hypoplasia (FDH), an X-linked dominant syndrome characterized by defects in mesodermal and endodermal tissues. PORCN is also an emerging target for cancer therapeutics. Despite the importance of this enzyme, its structure remains poorly understood. Recently, the crystal structure of DltB, an MBOAT family member from bacteria, was solved. In this report, we use experimental data along with homology modelling to DltB to determine the membrane topology of PORCN. Our studies reveal that PORCN has 11 membrane domains, comprising nine transmembrane spanning domains and two reentrant domains. The N-terminus is oriented towards the lumen while the C-terminus is oriented towards the cytosol. Like DltB, PORCN has a funnel-like structure that is encapsulated by multiple membrane-spanning helices. This new model for PORCN topology allows us to map residues that are important for biological activity (and implicated in FDH) onto its three-dimensional structure.

## Graphical abstract

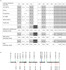

## 1. Background

Wnt gradients are essential for proper embryonic development and adult homeostasis in both invertebrate and vertebrate organisms. The formation of Wnt gradients requires the activity of Porcupine (PORCN), an O-acyl transferase that primarily resides in the endoplasmic reticulum (ER) [1,2]. PORCN was initially identified as a segment polarity gene in fruit flies; mutations lead to an accumulation of Wg/WNT1 protein at the site of synthesis [3]. A role for PORCN in Wnt trafficking, secretion and/or transport has been confirmed by numerous studies in vertebrates [4–12]. Loss-of-function studies indicate that PORCN is required for the trafficking of Wnt from the ER to the Golgi [5,8]. Therefore, in the absence of functional PORCN, Wnts are not trafficked to the cell surface nor are they subsequently secreted or distributed in gradients [3–8].

Bioinformatic analyses place PORCN in a large superfamily of membrane-bound O-acyl transferases (MBOATs) [1]. All MBOATs share two absolutely conserved residues, which correspond to N306 and H341 in mouse PORCN. In addition to the presence of multiple membrane-spanning domains, another unifying feature of MBOAT enzymes is that they promote the transfer of an acyl group from an acyl-coenzyme to a substrate. PORCN belongs to a subgroup of enzymes that specifically promote the acylation of protein or peptide substrates. Other relevant members of this subgroup include hedgehog acyl transferase (HHAT) and ghrelin O-acyl transferase (GOAT), which catalyse the lipid modification of Hedgehog and Ghrelin, respectively. Each of these enzymes catalyses reactions across the ER membrane. While the Acyl-CoA substrate is localized to the cytosol [13], the protein substrate (Wnt, Hh or Ghrelin) is located in the lumen of the ER [14,15]. Most MBOAT enzymes, including PORCN, HHAT and GOAT share a highly conserved histidine residue [1]. Though many reports indicate that the histidine residue is required for catalysis [1,16–20], subsequent data showing that one HHAT variant, with a mutated histidine, retains significant activity challenges this idea [21].

The only MBOAT for which the crystal structure has been reported is DltB, a bacterial enzyme that promotes the D-alanylation of cell wall teichoic acid [22]. The elucidated structure of DltB reveals that it is composed of a ring of 11 transmembrane domains, which forms a funnel embedded in the lipid bilayer [22]. The authors speculate that the funnel may be important for substrate binding or catalysis [22].

Biochemical studies have confirmed that PORCN catalyses the addition of a monounsaturated fatty acid, palmitoleate (C16:1), to a highly conserved serine residue corresponding to S224 in WNT1 [5,7,10,12,23,24]. Substitution of this conserved serine with an alanine or a cysteine abolishes Wnt palmit(e)oylation, secretion and signalling [5–7,10,25].

Additional studies specifically highlight the importance of the PORCN-dependent lipid modification. In addition to being required for Wnt gradient formation, PORCN-dependent lipid modifications are essential for binding of Wnts to Frizzled (FZD) receptors [26,27]. Hypomorphic or null alleles of PORCN cause segment polarity defects in flies [3], gastrulation arrest in mice [4,28] and focal dermal hypoplasia (FDH), a multisystem disorder in humans [29–34]. Because of its requirement for Wnt signalling, PORCN is actively being pursued as a therapeutic target in Wnt-driven cancers [35–43].

The existence of multiple PORCN mutations that cause FDH provide us with a rare opportunity to investigate the relationship between the structure and function of an MBOAT family member [29–34]. Although multiple groups have begun to address the structural features of PORCN, significant uncertainty about the membrane topology remains [25,29,36,44–46].

In this study, we used four distinct methods to characterize the membrane topology of PORCN. First, we leveraged topology modelling software to predict the location of transmembrane spanning domains. Second, we used a differential solubilization technique to distinguish whether different epitopes are oriented towards the ER lumen or cytosol. For these methods, we used an antibody against PORCN generated in our laboratory as well as commercially available antibodies that recognize multiple epitope tags. Third, we created PORCN variants with engineered consensus sites for the addition of N-linked glycosylation, which only occurs in the lumen of the ER. We then assessed the glycosylation status of different variants. Fourth, we used homology modelling and molecular dynamics simulation to predict the three-dimensional structure of PORCN. Taken together, these studies reveal that PORCN has nine transmembrane domains with two reentrant membrane domains.

## 2. Material and methods

p3xFLAG-CMV-14 (pCMV14/3xFLAG-Pax3 was a gift from Jonathan Epstein (Addgene plasmid no. 25427; http://n2t.net/addgene:25427; RRID: Addgene_25427) and wild-type/HA-tagged mouse and human PORCN variant D were kindly provided by Tatsui Kadowaki (Xi'an Jiaotong-Liverpool University) [2]. pHYK.Lysozyme-MYC was a gift from Hugh R. B. Pelham [47].

### 2.1. Materials

Materials and their respective vendors are as follows: DMEM, PBS, 200 mM l-Glutamine, 100× Penicillin/Streptomycin 0.25% Trypsin + 0.1%EDTA (Corning); Fetal Bovine Serum (Atlanta Biologicals); Fugene HD (Promega); Triton-X 100, DAPI (Roche); digitonin (ACROS); HEK 293T/17, COS-7, mouse anti-MYC 9E10.2 (conditioned media used at 1/10 dilution) (ATCC); 8-chamber glass slides, mouse anti-HA (1/2000 dilution) Immobilon-FL (Millipore), 16% paraformaldehyde (EMS); BSA, rabbit anti-FLAG (1/2000 dilution), DMSO, fatty acid free BSA, TCEP Tris(2-carboxyethyl) phosphine hydrochloride, TBTA Tris[(1-benzyl-1H-1,2,3-triazol-4-yl)methyl]amine, Copper Sulfate, NP-40 (Sigma); Alexa Fluor 647-AffiniPure Goat Anti-rabbit IgG (H + L) (1/200 dilution), Cy2 AffiniPure Donkey Anti-Mouse IgG (H + L) (1/200 dilution), anti-mouse Alkaline Phosphatase (H + L) (1/1500), anti-rabbit Alkaline Phosphatase (H + L) (1/1500) (Jackson ImmunoResearch); Mouse anti-GFP (1/15 000 dilution) (Clontech); SlowFade Gold antifade reagent (Thermo Scientific); Biotin Azide, Alexa Fluor_680 Goat Anti-mouse (1/4000 dilution), pcDNA3.1 (Invitrogen); IRDye800 conjugated streptavidin (1/5000 dilution), Odyssey blocking buffer in PBS (Licor); Protein A/G agarose beads, Halt protease and phosphatase inhibitor cocktail (Pierce); and Palmitic Acid Alkyne (Cayman Chemical).

### 2.2. Constructs

Chick Frizzled-10 MYC-His was created by cloning full-length cFz10 without the stop codon into pcDNA3.1/MYC-His(-)A.

Human PORCN variant D (hPORCND) was used to verify the specificity of the PORCN antibody. hPORCND was cloned into pcDNA3.1/MYC-His(-)A with the stop codon intact to prevent the addition of the MYC-His epitope [48]. HA-tagged hPORCND was created by replacing the 5′ end of hPORCND with the 5′ end of HA-tagged mouse PORCN variant D (mPORCND). The resulting protein has a 3x HA epitope tag, amino acid 2–114 mouse PORCN and amino acid 115–461 human PORCN. This was then cloned into pcDNA3.1/MYC-His(-)A with the stop codon intact to prevent the addition of the MYC-His epitope.

MPORCND was subcloned into p3xFLAG-CMV-14 to add the 3xFLAG epitope (DYKDHDGDYKDHDIDYKDDDDK) to the C-terminus (PORCN-3XFLAG) [48]. The MYC epitope (EQKLISEEDL) was added to the N-terminus of PORCN-3XFLAG and wild-type PORCN and the C-terminus of wild-type PORCN by PCR. All the internal MYC epitope tags with or without linkers were added to PORCN-3XFLAG in the p3xFLAG-CMV-14 vector using overlapping extension PCR (inserted after amino acid no. without linker: 47, 92, 118, 145, 181, 223, 289, 323, 376, 427; inserted after amino acid no. with linker SGGGGS added to each end of MYC epitope: 47, 69, 92, 343). All constructs lacking 3XFLAG tags were subcloned into pcDNA3.1.

For the glycosylation studies, we used HA-mPORCN cloned into pcDNA3.1/MYC-His(-)A (note: the stop codon was not removed, therefore HA-mPORCN did not get the MYC-His epitope tags from the vector) and added the glycosylation sites by mutating the amino acids by overlapping extension PCR (H53N, R90N, A134N, P184N, R235N, D283N, D384N, D423N, K438N). The C-terminus glycosylation site with the linker was added by PCR (gylNVTyv). In addition, we used eGFP cloned into pcDNA3.1/MYC-His(-)A (note: the stop codon was not removed, therefore eGFP did not receive the MYC-His tag).

The generation of spGFP:WNT1 (209–239):F_C_ is described by Miranda *et al*. [10].

### 2.3. Generation of anti-PORCN polyclonal antibody

Generation of polyclonal antibodies to human PORCN was done in a collaboration with Prosetta Biosciences. The peptide that was used as the immunogen correlates to amino acids 281–300. The sequence of the peptide was cEWDLTVSKPLNVELPRSMVE.

### 2.4. Topology prediction algorithms and visualization tools

We used the TOPCONS web server to predict the membrane topology of mPORCN variant D. We also used the ExPASY server to generate a Kyte and Doolittle hydropathy plot [49]. Lastly, we took advantage of the TOPO2 tool to assist with the visualization of topology models [50,51]. OCTOPUS, Philius, Polyphobius, SCAMPI, SPOCTOPUS and TOPCONS were used to predict the membrane topology of PORCN [51–56].

### 2.5. Cell culture

COS-7 and HEK293T cells were grown in standard media (DMEM with 10% fetal bovine serum, 4 mM l-Glutamine and 1× penicillin/streptomycin) on 100 mm plates in humidified incubators set to 10% CO_2_.

### 2.6. Validation of human PORCN antibodies

HEK293T cells were plated on a 12-well plate 1 day prior to transfection to yield 80–90% density at transfection. Cells were transfected with human PORCN, HA-human PORCN constructs or without DNA (control) using FUGENE HD according to the manufacturer's instructions. After transfection, the cells were incubated overnight, washed 1× with PBS supplemented with 1 mM MgCl_2_ and 136 mM CaCl_2_ and lysed in 200 µl of 100 mM Hepes pH 7.4 + 100 mM NaCL + 1% TX-100. Lysates were separated by SDS–PAGE and analysed by western blot using rabbit anti-PORCN or mouse anti-HA antibodies. Alkaline phosphatase-conjugated secondary antibodies were used for detection.

### 2.7. Differential solubilization

COS-7 cells were plated onto 24-well plates 1 day prior to transfection to yield 80–90% density at transfection. Cells were transfected with control pHYK.Lysozyme-MYC-KDEL, cFz10-MYC-His or PORCN MYC tags/FLAG tag constructs with FUGENE HD according to manufacturer's instructions. The cells were incubated overnight, split to 8-chamber glass slides and incubated overnight again. The cells were fixed in 4% paraformaldehyde in PBS for 10 min room temperature and then washed three times (5 min each) in PBS. For Triton-X permeabilization, the cells were incubated 5 min on ice in 0.25% Triton-X 100 in PBS. For digitonin permeabilization, the cells were incubated 5 min on ice in 50 µg per ml digitonin in PBS. From this point on the cells remained at room temperature. The cells were washed twice with PBS, blocked 30 min in 1% BSA in PBS, incubated 1 h with Rabbit anti-FLAG and Mouse anti-MYC or with anti-PORCN alone, washed 3 times with PBS 5 min per wash, blocked 15 min in 1% BSA in PBS, incubated with anti-rabbit 647, anti-mouse Cy2 and DAPI for 1 h, washed 3 times with PBS 5 min per wash, post fixed with 4% paraformaldehyde in PBS for 10 min, wash twice with PBS, mounted in SlowFade and imaged on a Zeiss LSM 710 with a 40× Oil objective (EC Plan-Neofluar 40×/1.3 oil). DAPI was visualized with the 405-nm laser, anti-mouse Cy2 was visualized with a 488-nm laser and anti-Rabbit 647 was visualized using the 633-nm laser. Z-stacks were collected and processed to generate maximum intensity projections. All images were analysed in Adobe Photoshop.

### 2.8. Click chemistry palmitoylation assay

HEK293T cells were seeded in a 6-well plate, incubated overnight and co-transfected with 1.65 µg of pcDNA3.1 encoding spGFP:WNT1 (209–239):F_C_ fusion along with 1.65 µg of pcDNA 3.1 encoding eGFP (as filler) or mPORCND tagged constructs using the FUGENE HD transfection kit (Promega) according to standard protocol. The cells were incubated overnight and then metabolically labelled with 100 µM of Alkyne palmitic acid (Alk-C16) or treated with DMSO as a control. After metabolic labelling of cells with Alk-C16 for 20–24 h, the cells were washed once with PBS and lysed in 300 µl of 100 mM Sodium Phosphate, pH 7.5 with 150 mM NaCl, 1% NP-40 and 1× Halt protease and phosphatase inhibitor cocktail for 1 h at 4°C. The lysate was cleared by centrifuging for 10 min and 260 µl of supernatant was transferred to a new tube. The F_C_ containing proteins were immunoprecipitated with Protein A/G beads. Proteins retained on the beads were subjected to click chemistry with biotin azide before separation by SDS–PAGE and analysis by western blot [11,12]. The blots were probed with IRDye800 conjugated streptavidin, and anti-GFP antibody followed by goat Alexa Fluor_680-conjugated anti-mouse secondary antibody. Blots were then scanned using an Odyssey CLx imaging system and analysed using Image Studio software.

### 2.9. Glycosylation analysis

HEK293T cells were split to 24-well plates 1 day prior to transfection to yield 80–90% confluence the next day. The cells were then transfected with the indicated construct with Fugene HD reagent according to the manufacturer's instructions. The cells were incubated for 4 h at 37°C 10% CO_2_. DMSO or tunicamycin was then added to yield a final tunicamycin concentration of 2.5 ng µl^−1^ (DMSO was diluted the same way as a control). The cells were incubated overnight, lysed in 150 µl of 100 mM Hepes pH7.4 + 100 mM NaCl + 1% TX-100, transferred to a microfuge tube and heated to 60°C for 5 min with SDS–PAGE loading dye (final concentration: 0.32 M Tris pH6.8 with 20 mM DTT, 2% SDS, 10% glycerol and 0.004% Bromophenol Blue) added. The lysates were then triturated with a 25-gauge needle five times, ran on a 10% SDS–PAGE gel and electroblotted to PVDF Immobilon-FL membrane. The blot was probed with anti-HA antibody followed by goat Alexa Fluor_680-conjugated anti-mouse secondary antibody. Blots were scanned in an Odyssey CLx imaging system and analysed using Image Studio software.

### 2.10. Homology modelling of PORCN

A homology model of mPORCND was generated using YASARA (v. 19.12.14) (Vienna, Austria) in automated mode [57], using the sequence of mouse PORCN isoform D (accession code, NP_076127.1). The model primarily used coordinates from the X-ray crystal structure of DltB, an integral membrane protein from *Streptococcus thermophiles* (PDB = 6BUH, solved to 3.15 Å) as a homology template [22]. YASARA created an ensemble of five models (6BUH-C01 through 6BUH-C05) with *Z*-scores ranging from −1.313 to −1.591. YASARA also created a hybrid model of the five individual models. However, the *Z*-score of the hybrid was less favourable (−1.840) than for model 6BUH-C01 (*Z*-score = −1.313). As such, model 6BUH-C01 was designated as the final model for mPORCND.

### 2.11. Molecular dynamics simulations

A molecular dynamics simulation of PORCN was carried out to determine the stability of the structure and to examine its equilibrated conformation using the Amber18 and AmberTools18 packages [58]. The protein was embedded into a simulated bilayer of 1-palmitoyl-2-oleoylphosphatidylcholine (POPC) molecules employing the CHARMM Membrane Builder tool [59]. Topology files for the protein were constructed in TLEAP [22] using the FF14SB [60], LIPID17 and GAFF force fields [61]. The membrane-embedded protein was solvated in explicit TIP3P water arranged in an 8 Å box from the surface of the protein–membrane complex [62]. Prior to minimization, equilibration and production run, the complexes were charge neutralized by the addition of chloride anions. The MD simulations were carried out using particle mesh Ewald (PME) graphical processor unit-accelerated MD with the PME MD Compute Unified Device Architecture module of Amber18 [63]. Initially, the protein was subjected to (i) minimization with the restraint of the protein for 1000 cycles; (ii) unrestrained minimization for 1000 cycles; (iii) equilibration while warming from 50 K to 300 K for 30 ps using a constant-volume periodic boundary condition; (iv) equilibration with the restraint of the protein at 310 K for 20 ps with a constant-pressure periodic boundary condition and using isotropic pressure scaling; and (v) unrestrained equilibration at 300 K for 500 ps. After minimization and equilibration, the protein was subjected to a production run at 300 K for 50 ns. Preparation steps 1 and 2 comprised 500 steps of steepest descent, followed by 500 steps of conjugate-gradient descent. Steps 3–5 and the production runs used Langevin temperature regulation with the collision frequency of 2.0 ps^−1^ [64], bonds involving hydrogen were constrained by the SHAKE algorithm [65]. A 12 Å cutoff was used for non-bonded interactions calculated by the PME method [66]. The MD simulations were monitored by examination of the internal energy and root mean standard deviation (RMSD) of the resulting trajectories (electronic supplementary material, figure S2). The production run trajectories were visualized using visual molecular dynamics (VMD; v.1.9.3) [67], while static snapshots of the protein were visualized using PYMOL (Schrödinger, San Diego, CA).

## 3. Results

### 3.1. Bioinformatic analysis of PORCN topology

**Figure 1.**
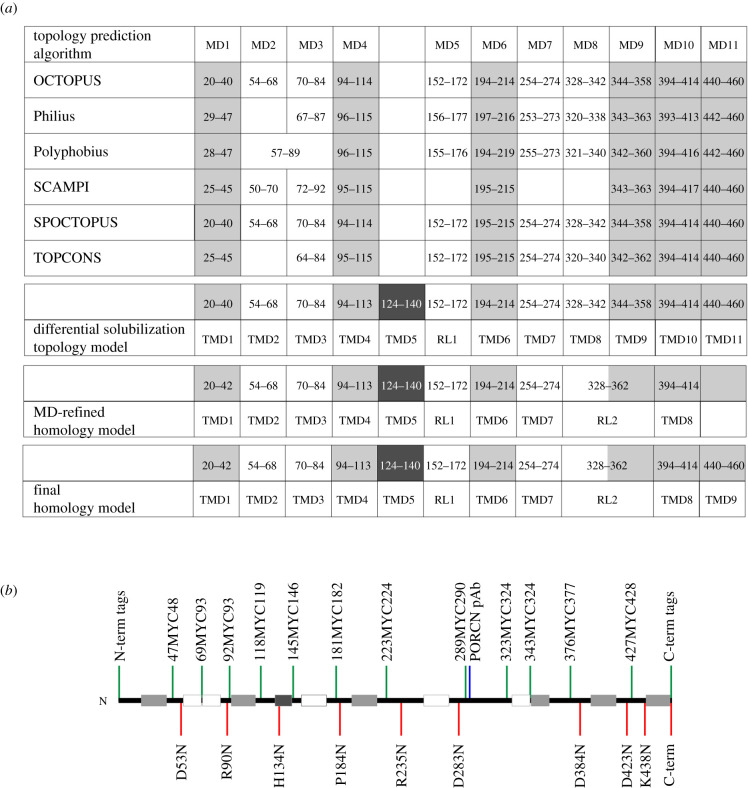
(*a*) Bioinformatic analysis of PORCN topology and final model. The mouse PORCN protein sequence was subjected to bioinformatics analysis to predict the number of transmembrane domains via TOPCONS website (http://topcons.cbr.su.se). Results were summarized in (*a*) upper table, in which 8–11 transmembrane domains were predicted. Light grey boxes indicated transmembrane domains that all algorithms predicted and white boxes for the domains that some of the algorithms predicted. The second table summarizes the number of transmembrane domains (11) and reentrant loops (1) as determined by differential solubilization experiments. The third table summarizes the number of transmembrane domains (8) and reentrant loops (2) as determined by homology modelling. The last table represents the final model that summarizes the differential solubilization and homology modelling results for the number of transmembrane domains (9) and reentrant loops (2). (*b*) Location of MYC and glycosylation tags in mouse PORCN with the transmembrane domains shown with light grey (consensus prediction), white (partial prediction) or dark grey boxes (not predicted).

As a first step towards understanding the membrane topology of PORCN, we used six different algorithms to predict the location and number of transmembrane domains (figure 1). There was excellent consensus around the existence, length and location of six membrane domains (figure 1, shown in grey). Although there was not a clear consensus about the total number of membrane domains, our synthesis of the different predictions suggests the presence of 11 membrane domains. Additionally, there was no clear consensus about the orientation of the amino and carboxy-termini of PORCN [51–56].

### 3.2. Validation of differential solubilization methodology

**Figure 2.**
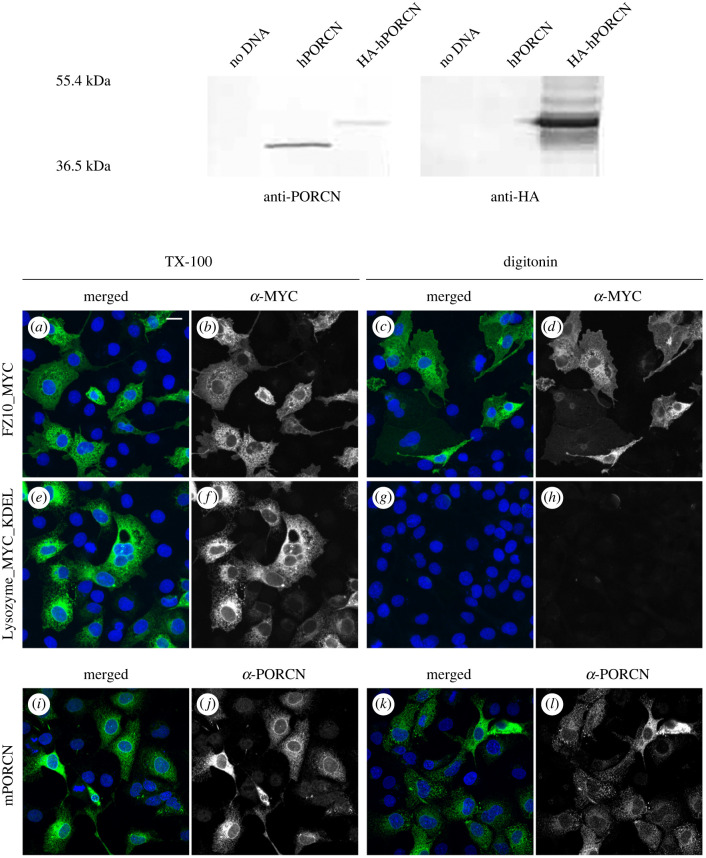
Differential solubilization to determine luminal and cytoplasmic loops and anti-PORCN loop located in the cytosol. HEK 293T cells were transfected with human PORCN, HA-human PORCN or no DNA (top panel). Lysates were analysed by western blot and probed with anti-PORCN or anti-HA. A distinct band is localized around 42 kDa for HA-human PORCN and 38 kDa for untagged human PORCN. COS7 cells were transfected with FZ10-MYC, Lysozyme-MYC-KDEL or mouse PORCN, fixed and subjected to either TX-100 or digitonin permeabilization followed by antibody detection (bottom panel). FZ10-MYC permeabilized with either TX-100 (*a*,*b*) or digitonin (*c*,*d*) immunostained with the anti-MYC antibody indicating that C-terminus was located in the cytosol. Lysozyme-MYC-KDEL, which has a KDEL sequence on the C-terminus to localized the protein in the endoplasmic reticulum (ER), permeabilized with TX-100 (*e*,*f*) had anti-MYC antibody immunostaining in the ER. When Lysozyme-MYC-KDEL was permeabilized with digitonin (*g*,*h*) little anti-MYC immunostaining was detected indicating that the protein was in the lumen of the ER. mPORCN when permeabilized with either TX-100 (*i*,*j*) or digitonin (*k*,*l*) immunostained with the anti-PORCN antibody indicating that the loop the antibody detects is in the cytosol. Scale bar is 20 µm.

We then sought to generate experimental evidence to support or refute the predictions from our bioinformatics analysis. To do this, we analysed wild-type and epitope-tagged variants of PORCN by differential solubilization [68–70]. For the differential solubilization method, fixed cells are solubilized with either TX-100 or digitonin before being subjected to immunostaining. Whereas TX-100 solubilizes both the plasma membrane and the ER membrane, digitonin only solubilizes the plasma membrane. In TX-100 cells, both cytosolic and luminal epitopes should be accessible to antibodies and thus, can be visualized by immunostaining. By contrast, in digitonin labelled cells, only epitopes that are oriented towards the cytosol should be accessible to antibodies. Because the cells were transiently transfected in this experiment, some cells received DNA while others did not. As such, the non-transfected cells (labelled with DAPI only) served as a control for nonspecific antibody staining. Examination of these cells reveals little to no nonspecific staining. To further validate this methodology, we tested the localization of the C-terminus of Frizzled10 (FZD10), which is a classic seven-transmembrane spanning protein resembling a G-protein coupled receptor, (figure 2, bottom panel, *a*–*d*) and Lysozyme-MYC-KDEL, an ER-resident protein (figure 2, bottom panel, *e*–*h*). As expected, our data show that the MYC epitope on the C-terminus of FZD10 was accessible to antibodies when cells were solubilized with either TX-100 or digitonin, thus localizing the C-terminus to the cytosol. Conversely, the MYC epitope on Lysozyme-MYC-KDEL was accessible when cells were solubilized in TX-100, but not digitonin, thus localizing it to the ER lumen. This important control was performed each time we carried out the differential solubilization experiment.

### 3.3. PORCN topology determination using an antibody against PORCN

As a first step towards understanding the membrane topology of PORCN, we first sought to raise polyclonal antibodies to peptides found in different predicted loops of hPORCND. Of the five peptides used as immunogens, only one, which spanned residues 281–300, successfully raised an immunogenic response. Purified antiserum was validated by western blot analysis with human PORCN and HA-human PORCN lysates isolated from transiently transfected HEK293T cells (figure 2, top panel; electronic supplementary material, figure S1*a*). Several nonspecific bands were detected in both control and experimental lysates, with the two most prominent bands migrating at 58 and 63 kDa. In addition, the anti-PORCN antibody specifically detects a band of approximately 38 and 42 kDa in the lysates from hPORCN and HA-hPORCN transfected cells, respectively. Similarly, the anti-HA antibody specifically detects a band of 42 kDa in lysates from the HA-hPORCN transfected cells. To further validate the anti-PORCN antibody for immunostaining, COS7 cells transiently transfected with mPORCN-MYC were fixed and solubilized with either TX-100 or digitonin, and then co-immunostained with anti-MYC and anti-PORCN antibodies (electronic supplementary material, figure S1*b*). The staining patterns generated by the two antibodies were indistinguishable, indicating that the anti-PORCN antibody faithfully replicates the staining pattern yielded by the anti-MYC antibody. In addition, the anti-PORCN antibody showed no detectable staining in non-transfected cells (DAPI labelling only), thus demonstrating the specificity of the anti-PORCN antibody for our differential solubilization studies (electronic supplementary material, figure S1*b*). We then used the purified antiserum to immunostain differentially solubilized COS7 cells, which were transiently transfected with wild-type PORCN (figure 2, bottom panel). Our data indicate that the anti-PORCN antibody was able to access the residues 281–300 in the presence of either TX-100 or digitonin. Thus, we conclude that this loop is oriented towards the cytosol (figure 2, bottom panel, *i*–*l*). This experiment was performed three times.

### 3.4. Biological activity of tagged PORCN constructs

**Figure 3.**
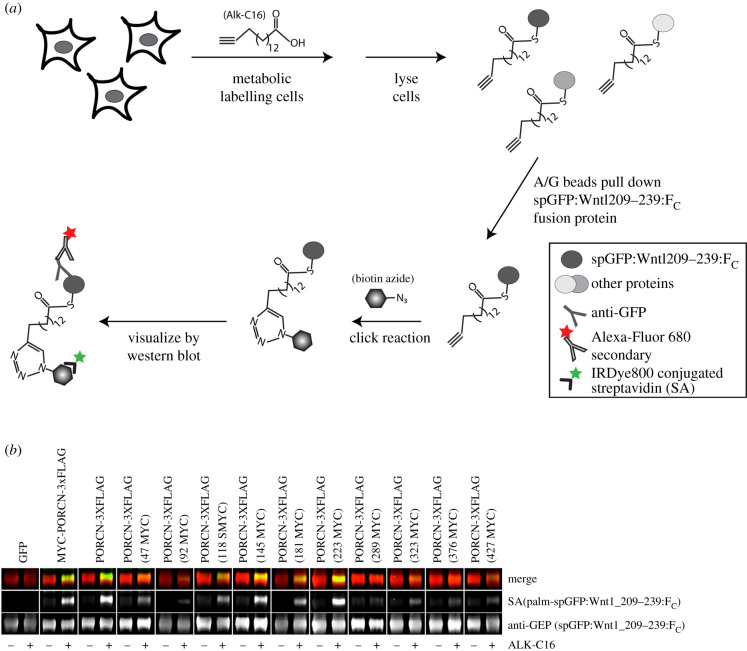
Biological activity of PORCN tagged with MYC and 3xFLAG. (*a*) A schematic of the click labelling reaction and detection is shown. (*b*) HEK 293T cells were transfected with the indicated PORCN tag and spGFP:WNT1 (209–239):F_C_ (a known PORCN substrate), metabolically labelled with Alkyne palmitic acid (Alk-C16), and lysed. spGFP:WNT1 (209–239):F_C_ was then precipitated with A/G agarose beads, subjected to click chemistry and analysed on a western blot. The blots were probed with IRDye800 conjugated streptavidin, and anti-GFP antibody followed by goat Alexa Fluor_680-conjugated anti-mouse secondary antibody. All except 289MYC had detectable levels of activity. Each construct was tested at least twice.

Because we had only a single PORCN antibody, it was necessary to create a panel of epitope-tagged PORCN constructs to localize the termini and additional loops (figure 1*b*). As tagging proteins often affects the structure and function of the protein, we first tested the biological activity of our tagged variants using a click chemistry-based palmitoylation assay (figure 3,*a*) [11,12,71,72]. Activity in this assay is assessed by comparing bands obtained for a single construct in the presence and absence of alkyne-palmitate (Alk-C16). Our results show that PORCN, bearing an amino MYC with carboxy-terminal tag (MYC-PORCN-3xFLAG) or carboxy-terminal (PORCN-3XFLAG) tag, is biologically active (figure 3*b*). We also show that PORCN-3XFLAG constructs with internal MYC tags introduced at residues 47, 118, 145, 181, 223, 323, 376 and 427 all retained appreciable activity, with 145 and 223 reliably having the most activity. The construct with the MYC tag inserted at residue 289 was the only one that lacked detectable activity. Notably, analysis of PORCN with a MYC tag inserted at residue 92 showed a faint band in the lane with Alk-C16, but not without Alk-C16. As such, we concluded that this construct maintained some activity.

### 3.5. Determination of the orientation of the amino and carboxy-termini of PORCN

**Figure 4.**
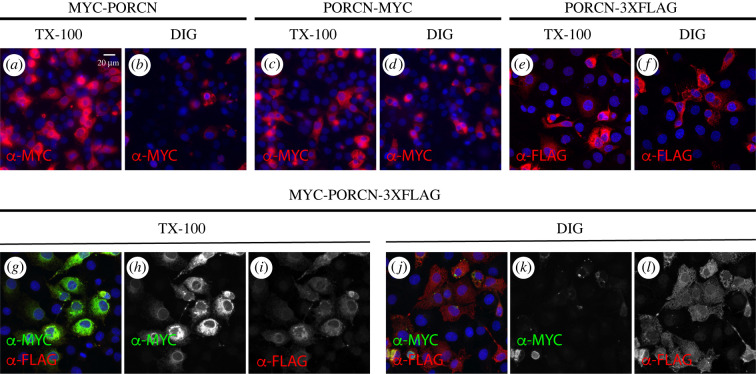
N-terminus of PORCN is in the lumen and the C-terminus is in the cytosol. We first tested the single tag MYC PORCN constructs tagged on either the N-terminus (*a*,*b*) or the C-terminus (*c*,*d*). While the C-terminus had immunostaining in both the TX-100 and digitonin permeabilization, the N-terminus did not, indicating that the C-terminus is localized to the cytosol and the N-terminus in the lumen. We also wanted to create an internal control for the internal MYC tags. Therefore, we replaced the C-Terminus MYC with 3x-FLAG (*e*,*f*) and it also immunostained when digitonin permeabilization was used. Finally, a MYC-PORCN-3xFLAG construct was tested (*g*–*l*) and repeated the result that the N-terminus is in the lumen and the C-terminus is in the cytosol. Each construct was tested at least twice. Scale bar is 20 µm.

We then sought to define the orientation of the N- and C-termini of PORCN. We first introduced a MYC tag onto the N- and C-termini of PORCN. When overexpressed in COS7 cells, the N-terminal MYC epitope was readily accessible to antibodies when cells were solubilized with TX-100 (figure 4*a*). However, upon solubilization with digitonin, the epitope was largely masked (figure 4*b*). Though some immunostaining was evident, it was not in the reticulated pattern of the ER. Thus, it seems likely that the N-terminus is oriented towards the lumen. By contrast, the C-terminally tagged PORCN was visualized when cells were solubilized with either TX-100 or digitonin (figure 4*c*,*d*), thus suggesting that the C-terminus is oriented towards the cytosol. Because different tags can sometimes yield different results, we also tagged PORCN on the C-terminus with 3XFLAG (in lieu of MYC). As before, immunostaining was evident in both TX-100 and digitonin labelled cells (figure 4*e*,*f*). Lastly, we assessed the localization of the N-terminus in PORCN with dual tags, a MYC tag on the N-terminus and the 3XFLAG tag on the C-terminus. Again, we were able to visualize staining for the MYC tag in TX-100, but not digitonin, solubilized cells (figure 4*h*,*k*). The 3XFLAG epitope was successfully stained in both TX-100 and digitonin solubilized cells (figure 4*i*,*l*). Thus, we conclude that the N-terminus is oriented towards the lumen and the C-terminus is oriented towards the cytosol. We further conclude that the use of a dual tag system does not profoundly alter the topology of PORCN.

### 3.6. Determination of the orientation of internal PORCN epitope tags

**Figure 5.**
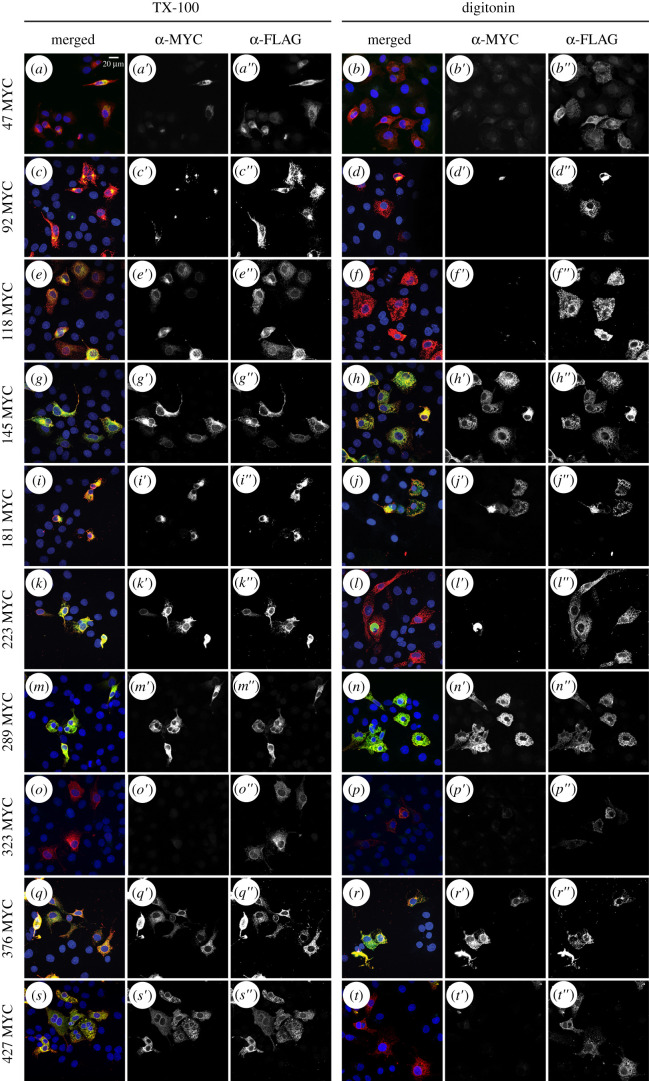
Determination of internal MYC tagged loops. All internal MYC constructs with C-terminus 3xFLAG were transfected into COS7 cells. The C-terminus 3xFLAG served as an internal control to ensure the topology was not altered by the internal MYC tag. As such, we expected the 3xFLAG epitope to immunostain with both the TX-100 and digitonin permeabilization for all the constructs, which was visualized (*b″*–*t″*). Immunostaining of the internal MYC with the TX-100 permeabilization was expected to yield the same immunostaining as the 3xFLAG epitope. However, this was not the case; 47MYC (*a′*), 92MYC (*c′*) and 323MYC (*o′*) did not have the same immunostaining pattern as the 3xFLAG, therefore we concluded that we could not use these three tags for a diagnostic of where the loop was located. As for the other constructs permeabilized with digitonin and immunostained with anti-MYC, 145MYC (*h′*), 181MYC (*j′*), 289MYC (*n′*) and 376MYC (*r′*) all showed the same immunostaining as 3xFLAG indicating that those loops are in the cytosol, while 118MYC (*f′*), 223MYC (*l′*), 427MYC (*t′*) did not have the same immunostaining pattern and therefore indicates that these loops are in the lumen. Each construct was tested at least twice. Scale bar is 20 µm.

PORCN variants with C-terminal 3XFLAG tags and internal MYC tags were then used to investigate the orientation of predicted internal loops using the differential solubilization assay. As a first step, we sought to validate that both epitopes were available when cells were solubilized with TX-100. The MYC epitopes for the fusions at positions 118, 145, 181, 223, 289, 376 and 427 (figure 5*e–e′*,*g–g′*,*i–i′*,*k–k′*,*m–m′*,*q–q′*,*s–s′*) were successfully visualized when cells were solubilized with TX-100. The results with digitonin solubilized cells suggest that the loops containing residues 118, 223 and 427 (figure 5*f′*,*l′*,*t′*) are oriented towards the lumen while loops containing residues 145, 181, 289 and 376 (figure 5*h′*,*j′*,*n′*,*r′*) are oriented towards the cytoplasm. As the fusions in which MYC tags were inserted at residues 47, 92 and 323 (figure 5*a–a′*,*c–c′*,*o–o′*) were not consistently immunostained when cells were solubilized with TX-100, the results from digitonin solubilized cells for these three constructs did not provide meaningful information.

**Figure 6.**
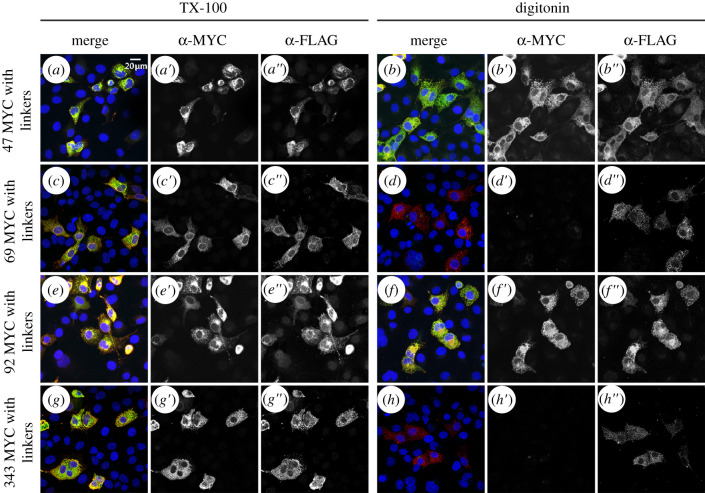
Determination of four additional internal MYC tagged loops with flexible linker. Four additional internal MYC constructs were created with a flexible linker to further assess the topology of PORCN. The four MYC constructs with C-terminus 3xFLAG were transfected into COS7 cells. The C-terminus 3xFLAG served as an internal control to ensure the topology was not altered by the internal MYC tag. As such, we expected the 3xFLAG epitope to immunostain with both the TX-100 and digitonin permeabilization for all the constructs, which was visualized (*a″*–*h″*). Immunostaining of the internal MYC with the TX-100 permeabilization was expected to yield the same immunostaining as the 3xFLAG epitope, which it did (*a′*,*c′*,*e′*,*g′*). Permeabilized with digitonin and immunostained with anti-MYC, 47MYC (*b′*) and 92MYC (*f′*) showed the same immunostaining as 3xFLAG indicating that those loops are in the cytosol, while 69MYC (*d′*) and 343MYC (*h′*) did not have the same immunostaining pattern and therefore indicates that these loops are in the lumen. Each construct was tested at least twice. Scale bar is 20 µm.

To circumvent the possibility that the epitopes for 47 and 92 were near the membrane bilayer or constrained in an unfavourable conformation, we then transfected and immunostained cells expressing MYC tags flanked by flexible linkers in the same positions. This time, both the MYC and 3XFLAG tags were detected when TX-100 solubilized cells were immunostained (figure 6*a′*,*e′*). Data from digitonin solubilized cells suggest that both of these epitopes are oriented towards the cytoplasm (figure 6*b′*,*f′*).

To test for the existence of additional short loops predicted between residues 68–70 (OCTOPUS, SCAMPI and SPOCTOPUS) and 342–344 (OCTOPUS, Philius, Polyphobius, SPOCTOPUS and TOPCONS), we created two more MYC epitope constructs flanked with flexible linkers at position 69 or 343. Both the MYC and 3XFLAG tags were detected upon immunostaining of TX-100 solubilized cells (figure 6*c′*,*g′*). Digitonin solubilized cells showed no detectable immunostaining, thus indicating that these epitopes are oriented towards the lumen (figure 6*d*’,*h*’).

To our surprise, our data for one of the loops (114–151) yielded different results depending on which construct was used. When the MYC tag was inserted into PORCN-3XFLAG at residue 118, the epitope localized to the lumen. However, when the MYC tag was inserted at position 145, the epitope was localized to the cytoplasm. These data suggested the possibility that there could be an additional membrane-spanning region that was not identified by the topology algorithms.

### 3.7. PORCN hydropathy plot suggests one additional membrane-spanning domain

**Figure 7.**
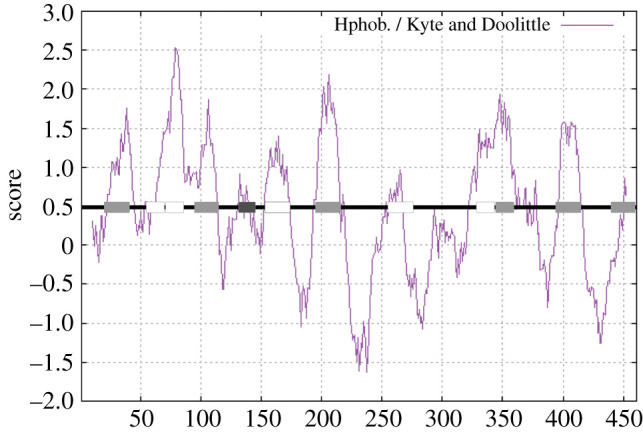
Hydropathy plot reveals one additional transmembrane domain. Kyte and Doolittle hydropathy plot was created for mouse PORCN. Overlaid is a schematic of PORCN with the boxes illustrating the predicted transmembrane domains. Light grey boxes were used for the domains that all six of the transmembrane prediction algorithms predicted, white boxes for the domains that were predicted by some of the algorithms and finally one dark grey box that none of the algorithms predicted by but do have a hydrophobic region indicated in the hydropathy plot.

Because we had conflicting data for one of the predicted loops of PORCN, we created a schematic in which we overlaid the membrane domains predicted by bioinformatics analysis along with the orientation of different loops onto a Kyte and Doolittle hydropathy plot (figure 7). As expected, the membrane domains predicted by bioinformatics analysis overlapped with hydrophobic regions on the plot. We noted, however, the existence of one additional moderately hydrophobic region spanning residues 124–140 (figure 7, dark grey box), which was not identified by any of the six algorithms used for our bioinformatics analyses. Because this domain is flanked by sequences which we have experimentally shown to be on opposite sides of the membrane, the most parsimonious interpretation is that residues 124–170 do indeed represent a previously unidentified membrane-spanning domain. Thus, these data initially suggested to us that PORCN has a total of 12 membrane domains (figure 1).

### 3.8. Analysis of membrane topology using *N*-glycosylation consensus sequence tags

We then sought to use an independent mechanism to further assess the orientation of different domains. To do this, we created 10 variants in which consensus sites for *N*-glycosylation were added (NxT/S) to HA-PORCN (figure 1*b*). For the internal sites, we mutated codons for residues that preceded X and T/S residues such that they now coded for an arginine (N). For the C-terminal site, we added an NVT sequence with linkers flanking the glycosylation site at the C-terminus of PORCN.

**Figure 8.**
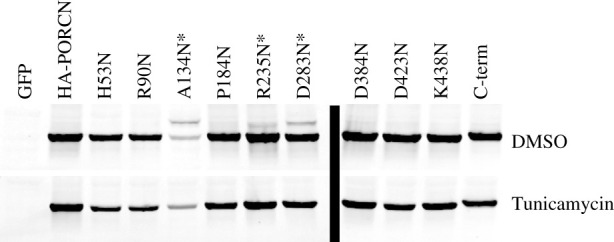
Analysis of PORCN glycosylation after the introduction of consensus sequences for *N*-glycosylation. HEK 293T cells were transfected with the HA-PORCN glycosylation site constructs as indicated. The cells were transfected for 4 h and then changed to media containing 2.5 ng µl^−1^ of tunicamycin or DMSO at the same concentration as a control. The cells were then lysed the next day and analysed by western blot. The fluorescent blot was inverted in Adobe Photoshop. HA-PORCN migrates as a single band of 42 kDa, three of the variants, A134N, R235N and D283N, migrated as a doublet, consistent with the addition of N-linked sugars (top row). When the cells were treated with tunicamycin, an inhibitor of glycosylation, the doublet was condensed into a single band (bottom row), further illustrating the upper band was due to glycosylation. With only one exception, each construct was tested at least twice for glycosylation in the absence and presence of tunicamycin. The R235N construct was treated with tunicamycin only once.

We then compared the migration of these variants to HA-PORCN using SDS–PAGE. Seven of the variants migrated as a single band of 42 kDa, suggesting that they were not glycosylated. This result, however, is not diagnostic as the epitope may not have been available for glycosylation. Three of the variants, A134N, R235N and D283N, migrated as a doublet, consistent with the addition of N-linked sugars (figure 8). To verify that the slower migrating band was indeed a consequence of N-linked glycosylation, we treated the cells with tunicamycin and showed that HA-PORCN variant was reduced to a single band (figure 8). While the data showing that R235 is glycosylated is consistent with the data from our differential solubilization studies, the finding that A134 and D283 are glycosylated is not in agreement with the prediction that A134 is in a transmembrane spanning domain and D283 is in the cytosol. As such, we then turned to homology modelling and molecular dynamics simulations to resolve these inconsistencies.

### 3.9. Homology modelling and molecular dynamics simulations of PORCN predict topology that aligns with differential solubilization data

**Figure 9.**
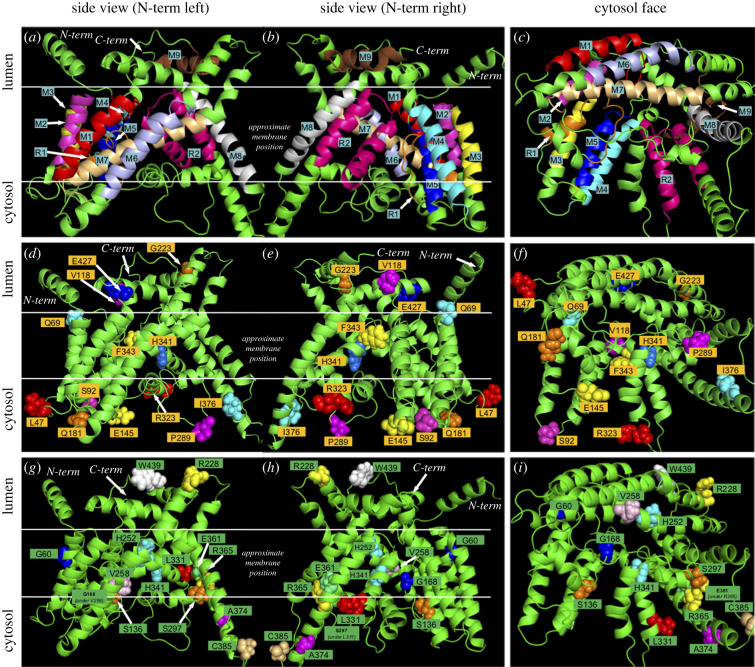
MD-refined homology model of mPORCND with key residues studied in epitope tagging experiments shown in spherical form and labelled. (*a*,*d*,*g*) Model positioned with the N-terminus on the left. (*b*,*e*,*h*) Model positioned with the N-terminus on the right. (*c*,*f*,*i*) Cytosolic face is shown, illustrating the funnel formation through the lipid bilayer. The top row shows the membrane alpha-helices colour labelled and numbered. Note that homology modelling does not place M9 (brown) in the membrane, but our final model does. The middle row shows labels for all of the internal MYC tags and the bottom row labels known human focal dermal hypoplasia (FDH) missense mutation sites.

**Figure 10.**
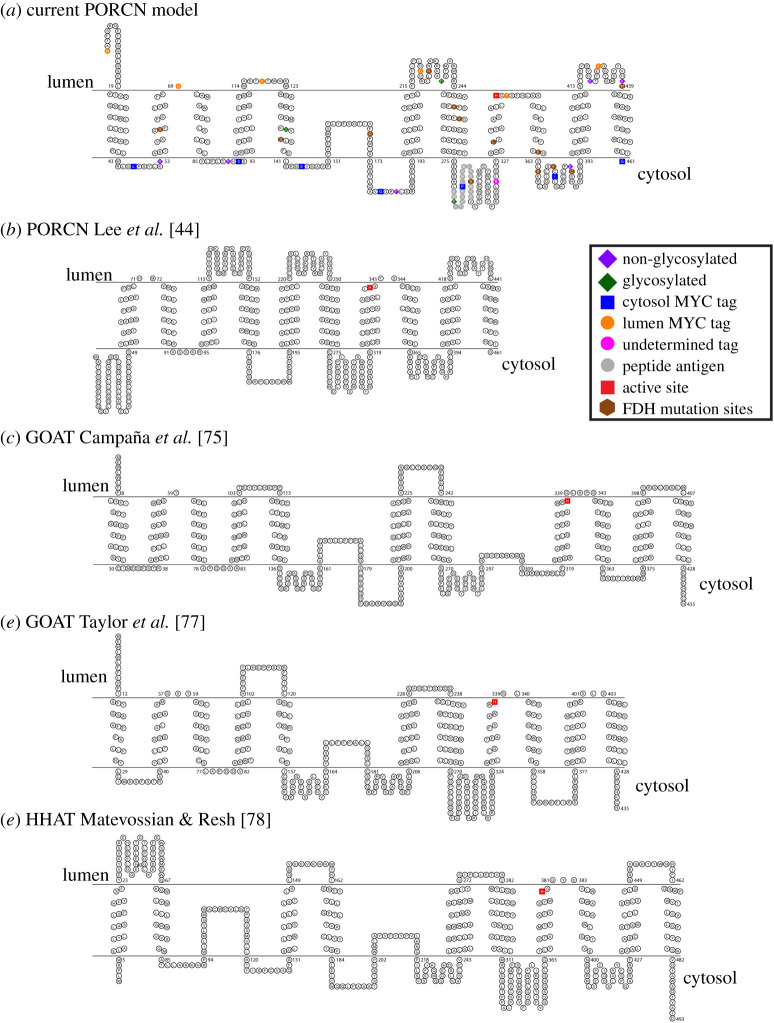
PORCN experimentally determined topology is similar to HHAT and GOAT topology. TOPO2 was used to create the topology of mouse PORCN that match the experimental data collected and homology modelling. The topologies for different reported models of PORCN (*b*), GOAT (*c*,*d*) and HHAT(*e*) are also shown for comparison [44,75,77,78].

A homology model of mPORCND was generated based on the high-resolution X-ray crystal structure of DltB, an MBOAT family member from *S. thermophiles,* which was solved at 2.5 Å (PDB = 4EZD; [22]. It is important to note that mPORCND and DltB are only 15% identical and 36% similar using the BLOSUM62 alignment matrix [73]. However, this is not unexpected as it has been established that membrane proteins are dramatically less well conserved than water-soluble proteins even though the functions, and presumably the structures, are well conserved [74]. Homology modelling has also been used to validate the structure of GOAT, which shares only 12% identity and 26% similarity with DltB (see figure 10*c*) [75]. However, Campaña *et al*. [75] only used regions sharing substantial homology between GOAT and DltB for the pairwise structural alignments. In addition to the primary template, the homology modeller in YASARA employed an ensemble of 137 proteins to model various domains of the final structure. We then carried out a molecular dynamics (MD) simulation to refine the homology model of PORCN under equilibrating conditions within a bi-lipid membrane. The protein produced from homology modelling was embedded into a simulated membrane comprised POPC molecules using the CHARMM membrane builder utility [59], with the orientation and position of the protein within the lipid determined using the automated PPM server [76]. The lipid–protein complex was subjected to a 50 ns production run at 300 K. We observed that the majority of the equilibration was complete within 25 ns for the membrane–protein complex, although the simulation was continued for a total of 50 ns (electronic supplementary material, figure S2). The root-mean-square deviation at equilibrium suggested it was stabilized at approximately 2.80 Å. The MD-refined homology model is depicted in figure 9, with the approximate membrane position as was determined by the PPM server during the initial membrane embedding step. Consistent with the crystal structure of DltB, the homology modelling clearly shows the presence of a funnel composed of a ring of 10 membrane domains, including eight transmembrane domains and two reentrant membrane domains. This funnel is thought to serve as the conduit for acyl-CoA to be translocated from the cytoplasmic side of the endoplasmic reticulum to the luminal side, where neonascent Wnts are located [14,22,75].

We then compared the position of N- and C-termini, and key amino acids observed in the homology model, with the position of the analogous features in MYC tagged experimental model (table 1). The MD-refined homology model predicts Q69, V118, G223 and E427 in the lumen, and L47, S92, E145, Q181, P289, R323 and I376 extending into the cytosolic space. F343 is predicted to reside in the membrane-spanning domain. Furthermore, the MD-refined homology model suggests that both the N- and C-termini reside in the lumen.

**Table 1.** Summary of molecular dynamics-comparing refined homology model and experimentally derived model for residues that were MYC tagged.

MYC tagged location	location in MD-refined homology model^a^	location in experimentally derived model^a^
N-terminus	L	L
L47	C	C
Q69	L	L
S92	C	C
V118	L	L
E145	C	C
Q181	C	C
G223	L	L
P289	C	C
R323	C	U
F343	M	L
I376	C	C
E427	L	L
C-terminus	L	C

^a^L, lumen; C, cytosol; M, membrane; U, undetermined.

The MD-refined homology model is remarkably consistent with our differential solubilization data, but not the glycosylation variant data. Specifically, the MD-refined homology model and the differential solubilization data are in full agreement with respect to the localization of the N-terminus and residues L47, Q69, S92, V118, E145, Q181, G223, P289, I376 and E427. One minor inconsistency is that the differential solubilization data indicate that F343 is oriented in the lumen while the MD-refined homology model indicates that it is positioned towards the luminal side of the membrane bilayer (table 1). However, it is important to note that the F343 MYC-tagged variant has flexible linkers on each side of the MYC tag (figure 6) and thus it is possible that these extra sequences may have caused the MYC tag to protrude out of the membrane bilayer into the lumen. The only significant discrepancy between the differential solubilization data and the MD-refined homology model is in the position of the C-terminus (figures 4 and 9). Whereas the homology model places the C-terminus in the lumen, the differential solubilization data indicate that it resides in the cytosol (figures 4 and 9). It is important to note that the C-terminus of PORCN extends beyond the sequences in DltB. As such, the homology model had no basis for assigning the C-terminus of PORCN to the cytosol or the lumen. As the differential solubilization data with both MYC and 3XFLAG tagged PORCN clearly show that the C-terminus is oriented towards the cytosol, we favour the idea that the C-terminus is indeed localized to the cytosol (figure 4).

### 3.10. Final model for PORCN membrane topology

We then used a combination of our differential solubilization data and our MD-refined homology modelling data to arrive at our final model for PORCN topology. Our final model for PORCN has nine transmembrane domains and two reentrant membrane domains (figure 1*a* and 10). We further propose that the N-terminus is oriented towards the lumen while the C-terminus is oriented towards the cytosol. The conserved N306 residue is localized to a cytoplasmic loop while the conserved H341 residue is positioned near the apex of the funnel.

## 4. Discussion

### 4.1. Summary of topology

Our studies using both experimental and computational approaches have provided us with a reliable membrane topology for PORCN that will undoubtedly advance the design of novel PORCN inhibitors and allow for deeper inquiry into the structure and function of this critical enzyme. Though we had set out to exclusively use anti-PORCN antibodies developed against different regions of PORCN for our experimental studies, only one of our PORCN peptide immunogens yielded usable antibodies. As such, we introduced multiple epitope tags throughout the protein in positions that were suggested to be located in the loops between the membrane domains. Because epitope tags are known to cause changes in protein structure, we were very circumspect about these studies. However, several pieces of data served to increase our confidence. First, the introduction of internal MYC tags never altered the position of our C-terminal 3xFLAG tag (figures 5 and 6). Second, the anti-PORCN antibody showed the same localization as the 289MYC tag (figure 5). And lastly, our structural modelling experiments in which we modelled PORCN based on the crystal structure of DltB validated all of our experimental data using epitope tags, except at the C-terminus. Recall that PORCN is significantly longer than DltB; as such, the modelling software had no basis for predicting the position of C-terminus of PORCN. Our final PORCN model indicates the presence of nine transmembrane domains and two reentrant membrane domains. As reentrant membrane domains are common in transmembrane proteins that are pores or channels, the presence of two reentrant membrane domains in PORCN is consistent with the presence of a funnel that shuttles palmitoleoyl-CoA from the cytoplasm to the lumen [79].

Interestingly, the introduction of consensus sites for the addition of N-linked glycosylation at 10 different positions yielded somewhat inconsistent results. Specifically, our differential solubilization and homology modelling data suggest that A134 is positioned in the membrane bilayer while D283 is localized in the cytosol. Notably, the differential solubilization data placing D283 in the cytosol is our strongest data as we were able to use anti-PORCN antibodies to define the orientation of wild-type (untagged) PORCN for that particular loop. However, our glycosylation data indicate that A134 and D283 are localized to the lumen. Taken together, these results provide more confidence in the differential solubilization data coupled with the MD-refined Homology model than in the results from studies in which we inserted new glycosylation sites. As such, we recommend that previous studies having relied on the insertion of glycosylation sites to provide insights about membrane topology be interpreted with caution. Though it is not clear why this method yields inconsistent results, one could speculate that the glycosylation of this site occurred while PORCN was still being translated and inserted into the membrane. Having said that, we cannot completely rule out the possibility that the low levels of homology between PORCN and DltB compromised the validity of the homology modelling and that PORCN has alternate conformers.

### 4.2. Comparison of our model with other PORCN models

Though our PORCN topology model is similar to other reported models in many respects, there are clear differences. Whereas we demonstrate the presence of nine transmembrane domains with two reentrant membrane domains, other models propose anywhere between 8 and 11 transmembrane domains [25,29,36,44–46]. And while our studies clearly indicate that the N-terminus is in the lumen, all four of the previous models show it in the cytoplasm. On the other hand, three of the four previous models agree with us that the C-terminus in the cytoplasm. Important experimental differences between our study and previous studies are that (i) we used a combination of experimental data and homology modelling and (ii) we created and used more epitope tags than any of the previous reports. The addition of additional epitope tags allowed us to identify a transmembrane domain that had not been predicted by any of the topology prediction algorithms.

### 4.3. Topology comparisons reveal that PORCN has a similar topology to GOAT and HHAT

Because PORCN, GOAT and HHAT share common functions as O-acyl transferases, we predicted that the three enzymes would also share structural features. As such, we then compared our new topology model to those reported for GOAT and HHAT (figure 10) [75,77,78,80]. The N-termini of both PORCN and GOAT are oriented towards the lumen while that of HHAT is reported to be oriented towards the cytosol. Consistent with the notion that the catalytic domain includes a conserved histidine (H341 in PORCN), the membrane topologies of PORCN, GOAT and HHAT are most similar in the C-terminal halves of the proteins, with all three proteins having their C-termini localized to the cytosol. In addition, PORCN transmembrane domains 1–5, reentrant membrane domain 1 and transmembrane domains 6 and 7 roughly align with those of GOAT, with only the lengths of the loops varying [75,77]. Notably, the conserved histidine in PORCN, GOAT and HHAT is positioned in the membrane bilayer. However, our homology modelling data indicate that the residues immediately following the histidine remain embedded in the membrane while those for GOAT and HHAT are predicted to extend into the lumen. Our studies place the conserved asparagine residue (N306 in PORCN), which is hypothesized to be important for binding to acyl-CoA, to a cytoplasmic loop. This aligns well with the GOAT topology model from Taylor *et al*., who place the conserved asparagine in the cytosol, but diverges slightly from the model put forth by Campaña *et al*. who place it in the membrane bilayer (close to the cytosolic face) [75,77].

### 4.4. Many residues implicated in focal dermal hypoplasia are localized to the funnel

Because our MD-refined homology model showed close agreement with our differential solubilization data, we used it to assess the location of key residues in PORCN, for which missense mutations are known to cause FDH (figures 9*g*–*i*, 10*a*). The MD-refined homology model shows that many of the residues that are involved in FDH are localized to the funnel, including S136, G168, H252, V258, S297, L331, H341, E361 and R365. These data are consistent with those from Campaña *et al.* [75], who suggested that surface-exposed side chains in GOAT are less important than those within the interior of the enzyme. In addition, our model places the H341 residue, which has specifically been implicated in catalysis, near the apex of the funnel in the membrane bilayer [81]. The localization of many of the residues implicated in FDH to the funnel is also consistent with the previous studies suggesting that the funnel is important for shuttling acyl-CoA from the cytoplasmic face of the lipid bilayer to the lumen [13,22,75]. Cumulatively, these data are consistent with the importance of the funnel for the PORCN function.

### 4.5. Future directions

Our proposed topology also raises some interesting questions for future investigations. The presence of the funnel offers up a mechanism for transporting acyl-CoA to the active site, marked by H341. Given that H341 is embedded in the membrane, how do Wnts gain access to the active site? We and others have previously shown that the Wnt sequences immediately surrounding the palmitoylation site form a thumb-like projection that is held together by two disulfide bonds, with the palmit(e)oylated serine being localized to the distal-most aspect of the thumb [10,23,44]. Thus, the palmit(e)oylation site is part of a rigid structure that could be inserted into the membrane. We note that many of the amino acids flanking the serine have positively charged side groups and thus, speculate that this may promote interactions of the thumb with negatively charged phospholipid head groups. Are these positively charged amino acids critical for binding? In addition, although mouse PORCN is not palmitoylated, human PORCN has been shown to be palmitoylated on cysteine 187 [12]. What class of palmitoyl acyl transferases might be responsible for this modification? As our topology places this residue in a cytosolic loop, we speculate that a member of the DHHC family is likely to be responsible for this modification. The region of PORCN that has splice variants (A–D) is located between transmembrane domains 6 and 7 in a luminal loop [48]. What is the role of these splice variants? As it turns out, splice variant A, which is the shortest of the variants, appears to have approximately 50% less activity than B–D [46]. As such, it is possible that the composition or length of the variant is important for Wnt binding to PORCN.

Lastly, we believe that aspects of the MD-refined homology model could be validated or improved by computational modelling studies of known PORCN inhibitors such as LKG-974 [82], GNF-6231 [83] and characterized analogues of each. While we believe the C-terminal region of our homology model is mis-assigned to the lumen, we are more confident on the structure of the transmembrane alpha-helices and reentrant loops, due to successful localization of residues in the cytosol and lumen consistent with differential solubilization data. As such, we predict the central cavities in the cytosolic and luminal domains contain plausible binding sites for small molecule inhibitors. Once binding modes are hypothesized, binding affinities can be estimated using MM-GBSA and MM-PBSA methods [84], and can be compared with experimental inhibitory potency, over a series of compounds. We expect that reasonable inhibitor-bound structures could be further refined by MD to improve the structure of PORCN, and could also be used as a template for virtual screening experiments.

In sum, the determination of the PORCN membrane topology along with the identification of residues that are critical for PORCN function (from FDH) will allow us to further our understanding of the relationship between structure and function for this important enzyme. We look forward to better understanding the roles of these residues in PORCN function.

## Data accessibility

This article has no additional data.
